# CK1ε/SRSF10 axis regulates the alternative splicing of Bcl-x in lung cancer cells

**DOI:** 10.1016/j.jbc.2025.110508

**Published:** 2025-07-21

**Authors:** Qi Sun, Yun Tang, Lian Wang, Boxin Liu, Jianhong Xiao, Hanbin Wang, Shujing Lei, Yuexuan Chen, Yi Liu, Shanshan Liu, Xibao Zhao, Jitian Zhang, Desheng Lu

**Affiliations:** 1 Guangdong Provincial Key Laboratory of Regional Immunity and Disease, International Cancer Center, Marshall Laboratory of Biomedical Engineering, Department of Pharmacology, Shenzhen University Medical School, Shenzhen University, Shenzhen, Guangdong, China; 2 School of Pharmacy, Shenzhen University Medical School, Shenzhen University, Shenzhen, Guangdong, China; 3 Department of Surgery, The University of Hong Kong-Shenzhen Hospital, Shenzhen, Guangdong, China

**Keywords:** CK1ε, Bcl-x, alternative splicing, SRSF10, lung cancer

## Abstract

The dysregulation of Bcl-x alternative splicing is associated with tumor development and chemoresistance. However, the underlying molecular mechanisms of Bcl-x splicing are still not well-defined. Here, we demonstrated that casein kinase 1ε (CK1ε) was involved in the regulation of Bcl-x alternative splicing. Initially, we noted that SR3029, a specific CK1δ/ε inhibitor, effectively reduced the mRNA and protein expression of Bcl-xL and accompanied by an increase in the mRNA and protein levels of Bcl-xS in a dose-dependent manner. Overexpression of CK1ε decreased the ratio of Bcl-xS/Bcl-xL mRNA and protein compared to the control cells, while depletion of CK1ε leads to an increase in the ratio of Bcl-xS/Bcl-xL. The overexpression of CK1ε also abrogated the impact of serine/arginine-rich splicing factor 10 (SRSF10) knockdown on the ratio of Bcl-xS/Bcl-xL. Subsequently, CK1ε was found to interact with SRSF10 and phosphorylate SRSF10 at S23 and S133, which may be required for the binding of SRSF10 to the Bcl-xL mRNA. Furthermore, depletion of SRSF10 markedly promoted apoptosis and inhibited the viability, proliferation, and colony formation in lung cancer cells. CK1δ/ε inhibitor SR3029 could further enhance the effect of silencing SRSF10 on biological behavior. The xenograft model of lung cancer cells confirmed that pharmacological inhibition of CK1ε and the knockdown of SRSF10 synergistically inhibited tumor growth. Taken together, our results revealed a novel mechanism by which the CK1ε/SRSF10 axis regulates the alternative splicing of the Bcl-x precursor mRNA, which may be a potential therapeutic target for lung cancer.

Alternative RNA splicing is a critical process that contributes to the diversity and complexity at the transcriptome and proteome levels. The same precursor mRNA (pre-mRNA) can produce multiple protein isoforms with different biological properties through alternative splicing (1, 2). Pre-mRNA splicing is performed by the spliceosome, a macromolecular machinery containing five small ribonucleoprotein complexes (U1, U2, U4, U5, and U6 snRNPs) and a large number of regulatory proteins (3). Aberrant alternative splicing may cause the disruption of normal cellular function, and eventually lead to multiple diseases, including cancer. Cancer cells may produce cancer-specific splicing variants which play an important role in tumorigenesis, progression, and chemoresistance (4, 5).

Bcl-x, a member of the Bcl-2 family with multiple BH3 domains, is a transmembrane (TM) protein in the mitochondria which plays a key role in the regulation of apoptosis (6, 7). Alternative splicing of exon 2 in Bcl-x pre-mRNA, either inclusion or exclusion of exon 2, results in two splice isoforms with opposing functions, a long antiapoptotic isoform Bcl-xL and a short proapoptotic Bcl-xS (6, 8). Bcl-xL contains C-terminal TM domain and four Bcl-2 homology domains (BH1-4), while Bcl-xS consists of BH3, BH4, and TM domains but lacking BH1 and BH2 domains (8, 9). The increased level of Bcl-xL has been frequently observed in various types of cancer and is considered as a driving force for apoptotic and chemotherapeutic resistance (10, 11). In contrast, the highly expressed Bcl-xS enhanced the sensitivity to apoptotic stimuli (7). Thus, switching splicing to the Bcl-xS isoform may have a multitude of benefits for the treatment of some cancers with Bcl-xL overexpression (8).

Multiple splicing factors and signaling components have been identified to influence the Bcl-xL/Bcl-xS splicing ratio, including serine/arginine-rich splicing factors (SRSFs) and heterogeneous nuclear ribonucleoproteins (hnRNPs) (12). SRSFs are a highly conserved family of RNA-binding proteins that share the RNA recognition motif domain at the N terminus and the arginine- and serine-rich region at the C terminus (13). This family consists of 12 members (SRSF1-12) that play an important role in pre-mRNA splicing and RNA metabolism (14). SRSF1 is the prototypical member of SR protein family and has been shown to facilitate the production of antiapoptotic splice variants of Bcl-x, leading to the inhibition of programmed cell death in breast cancer and chronic myeloid leukemia (12). Lv *et al.* reported that SRSF1 could inhibit autophagy through promoting the splicing of the long isoform of Bcl-x and interacting with PIK3C3 in lung cancer (15). Moreover, hnRNPA1 was found to enhance the splicing of the proapoptotic Bcl-xS variant by antagonizing SRSF1 activity (16). Choi *et al.* identified SRSF2 and SRSF6 as the modulatory factors of 5′ splice-site selection of Bcl-x pre-mRNA (17). Interestingly, DNA damage could abrogate the association of SRSF10 and hnRNPK with the Bcl-x pre-mRNA, and enhance the production of proapoptotic Bcl-xS in an ATM/CHK2-dependent manner (18). Additionally, the RNA-binding protein Sam68 was demonstrated to regulate the alternative splicing of Bcl-x. Sam68 could favor the selection of the upstream 5′ splice site of Bcl-x and the generation of the proapoptotic Bcl-xS isoform (19). Although SRSF proteins have been found to be overexpressed in several human cancers (20, 21, 22), the molecular mechanism by which SRSF proteins regulate Bcl-x alternative splicing remains to be further elucidated.

Casein kinase 1 (CK1) belongs to a family of serine/threonine (Ser/Thr) protein kinases consisting of six subtypes (α, δ, ε, γ1, γ2, and γ3), of which CK1δ and CK1ε have highly homologous kinase domains (23). CK1 regulates diverse cellular processes including DNA damage repair, circadian rhythm, apoptosis, and differentiation (23, 24, 25, 26, 27). Targeting CK1 family members has shown promise as a potential therapeutic strategy for various types of cancer. The specific CK1δ/ε inhibitor SR3029 could significantly suppress the occurrence and development of skin and colorectal tumors (28). SR3029 also inhibited the growth of breast tumors by inducing apoptosis of breast cancer cells (29). Our previous studies demonstrated that CK1δ/ε enhanced the SKP2-mediated degradation of amino-terminal enhancer of split (30). We further showed that CK1ε exhibited its oncogenic role in colorectal cancer by modulating the stability of Axin1 (31). However, it remains unknown whether CK1δ/ε is implicated in regulation of alternative splicing of pre-mRNA.

In our study, we observed that CK1δ/ε inhibitor SR3029 effectively reduced the expression of Bcl-xL mRNA while simultaneously enhancing the mRNA levels of Bcl-xS. We further explored the mechanism by which CK1ε mediated Bcl-x alternative splicing. Our results indicate that the CK1ε–SRSF10 axis regulates the alternative splicing of Bcl-x, and this axis is associated with the pathogenesis of lung cancer.

## Results

### CK1δ/ε inhibitor SR3029 regulates the alternative splicing of Bcl-x

**Figure 1:**
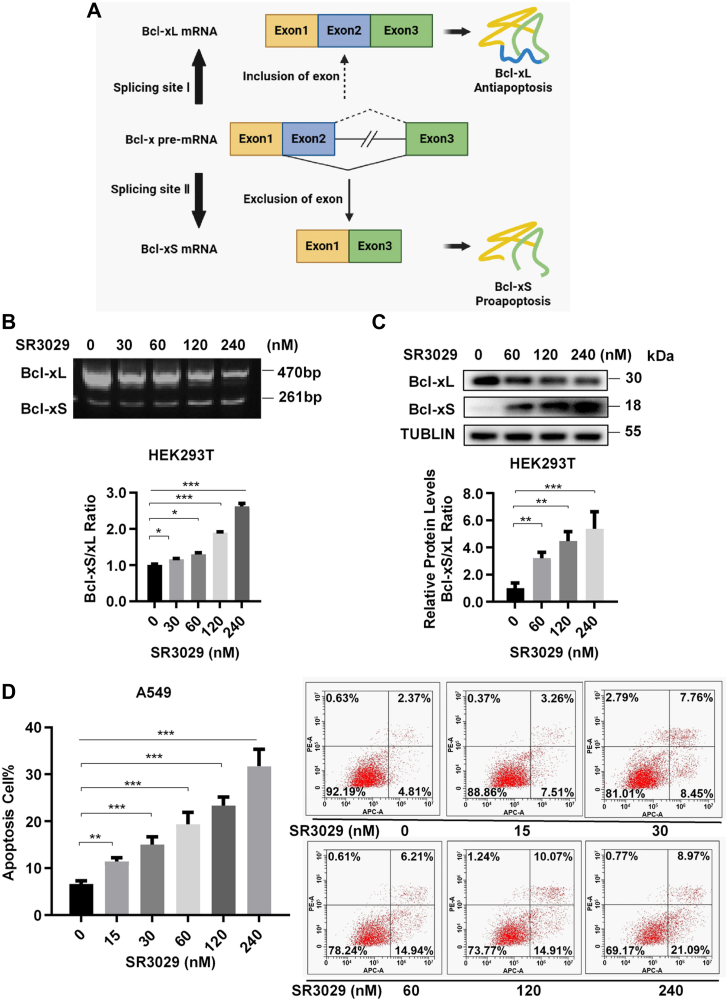
**CK1δ/ε inhibitor SR3029 mediates the alternative splicing of Bcl-x and exerts an apoptotic effect.***A*, schematic diagram of Bcl-x alternative splicing and the effects of different spliceosomes on apoptosis. *B*, after treating HEK293T cells with CK1δ/ε inhibitor SR3029 for 24 h, the effect of SR3029 on the expression of different Bcl-x splicing isomers was analyzed by reverse transcription-PCR (RT-PCR). *C*, after treating HEK293T cells with SR3029 at indicated concentrations for 24 h, the effect of SR3029 on protein expression of Bcl-xL and Bcl-xS was analyzed by Western blot. *D*, A549 cells were treated with dimethyl sulfoxide and different concentrations of SR3029 (0, 15, 30, 60, 120, and 240 nM) for 24 h, and apoptosis was detected by flow cytometry. Results are expressed as mean ± SD (n = 3, ∗*p* < 0.05, ∗∗*p* < 0.01, and ∗∗∗*p* < 0.001). CK1, casein kinase 1; HEK293T, human embryonic kidney 293T.

To identify novel small-molecule modulator of Bcl-x splicing, we conducted a kinase inhibitor library screen using real-time PCR in lung cancer A549 cells. After transcriptional processing, Bcl-x pre-mRNA produces two splicing isomers with different functions, proapoptotic Bcl-xS and antiapoptotic Bcl-xL, respectively (Fig. 1*A*). The primary screening identified that CK1δ/ε inhibitor SR3029 could regulate the alternative splicing of Bcl-x gene. To validate our finding, human embryonic kidney 293T (HEK293T) cells were treated with increasing concentrations of SR3029. Treatment with SR3029 dose dependently decreased the mRNA and protein levels of Bcl-xL with a concomitant increase in the mRNA and protein levels of Bcl-xS (Fig. 1, *B* and *C*). SR3029 also enhanced apoptosis in the same concentration range in A549 cells (Fig. 1*D*).

### CK1ε regulates the alternative splicing of Bcl-x

**Figure 2:**
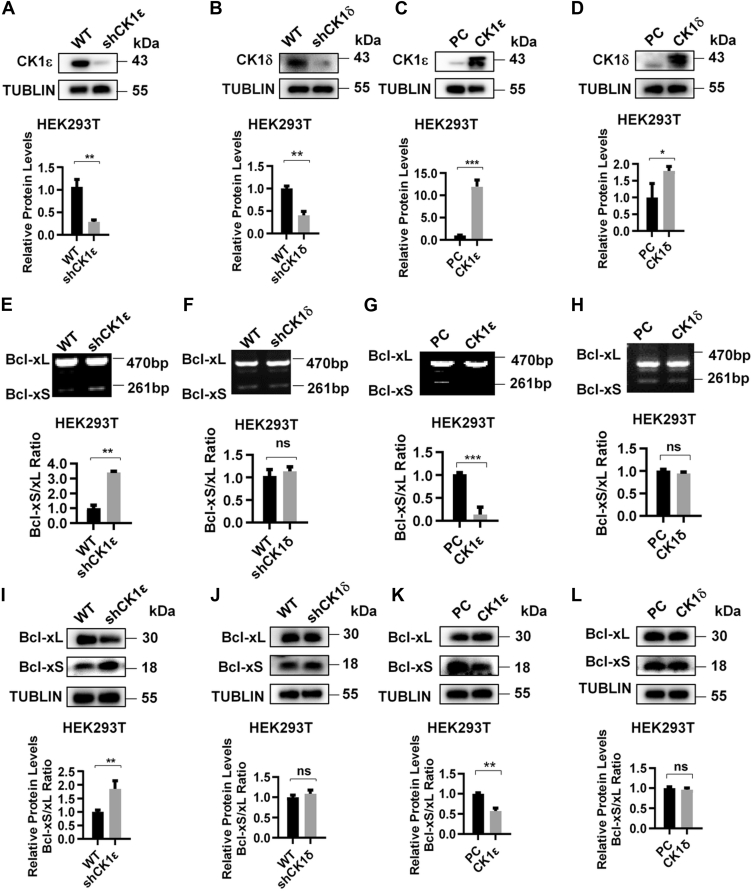
**CK1ε regulates the alternative splicing of Bcl-x in HEK293T cells.***A*–*D*, the effect of knockdown or overexpression of CK1ε and CK1δ was detected by Western blot. The protein quantitative results of the grayscale were shown below. *E* and *F*, the effect of CK1ε or CK1δ knockdown on Bcl-x alternative splicing was analyzed in HEK293T cells by RT-PCR. The quantitative results of the grayscale ratio of Bcl-xS to Bcl-xL are shown below. HEK293T cells were transfected with CK1ε (*G*) or CK1δ (*H*) expression plasmids for 48 h, and the effect of CK1δ/ε overexpression on Bcl-x alternative splicing was analyzed by RT-PCR. The quantitative results of the grayscale ratio of Bcl-xS to Bcl-xL are shown below. *I*–*L*, the effect of knockdown or overexpression of CK1ε and CK1δ on Bcl-xL and Bcl-xS protein levels was analyzed by Western blot. The protein quantitative results of the *grayscale* ratio of Bcl-xS to Bcl-xL were shown below. Results are expressed as mean ± SD (n = 3, ∗*p* < 0.05, ∗∗*p* < 0.01, and ∗∗∗*p* < 0.001). CK1, casein kinase 1; HEK293T, human embryonic kidney 293T.

**Figure 3:**
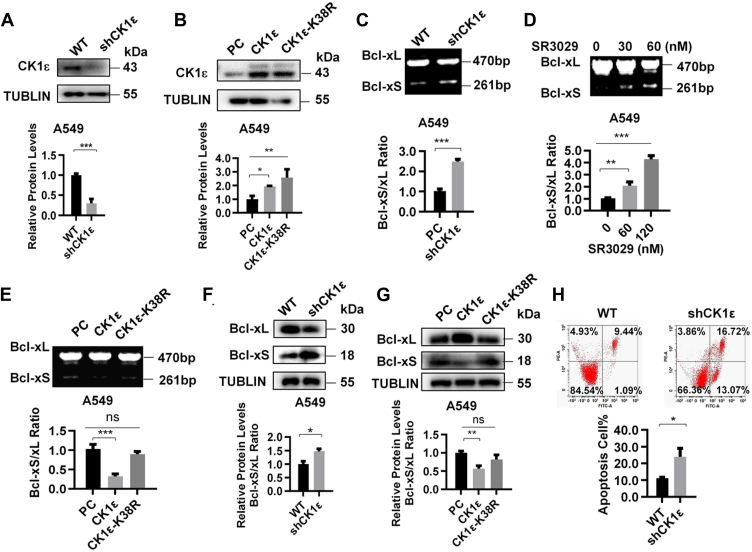
**CK1ε regulates the alternative splicing of Bcl-x in A549 cells.***A* and *B*, the effect of overexpression of CK1ε and CK1ε-K38R, and CK1ε knockdown in A549 cells was detected by Western blot. The protein quantitative results of the grayscale were shown below. *C*, the effect of endogenous CK1ε knockdown on the alternative splicing of Bcl-x was analyzed in A549 cells. The quantitative results of the grayscale ratio of Bcl-xS to Bcl-xL are shown below. *D*, A549 cells were treated with SR3029 for 24 h, and the effect of SR3029 on the expression of different Bcl-x splicing isomers was analyzed by RT-PCR. The quantitative results of the grayscale ratio of Bcl-xS to Bcl-xL are shown below. *E*, A549 cells were transfected with CK1ε or control vector and CK1ε-K38R (a kinase-dead mutant of CK1ε) expression plasmid. The expression of different Bcl-x splicing isomers was analyzed by RT-PCR. A representative gel was demonstrated, and the quantitative results of the grayscale ratio of Bcl-xS to Bcl-xL are shown below. *F*, the endogenous CK1ε was knockdown in A549 cells, and the protein levels of Bcl-xL and Bcl-xS were analyzed by Western blot. The quantitative results of the grayscale are shown below. *G*, A549 cells were transfected with CK1ε or control vector and CK1ε-K38R (a kinase-dead mutant of CK1ε) expression plasmid, and the protein levels of Bcl-xL and Bcl-xS were analyzed by Western blot. *H*, the endogenous CK1ε was knockdown in A549 cells, and apoptosis was detected by flow cytometry. Results are expressed as mean ± SD (n = 3, ∗*p* < 0.05, ∗∗*p* < 0.01, and ∗∗∗*p* < 0.001). CK1, casein kinase 1; HEK293T, human embryonic kidney 293T.

We next determined whether CK1δ/ε is implicated in the regulation of Bcl-x splicing. Lentiviral vector-mediated RNAi was used for silencing the expression of CK1δ or CK1ε gene in HEK293T cells. The protein expression of CK1ε or CK1δ was confirmed in cells with knockdown and overexpression of CK1ε or CK1δ using Western blot analysis (Fig. 2, *A*–*D*). Knockdown of CK1ε but not CK1δ significantly increased the ratio of Bcl-xS/Bcl-xL mRNA and protein compared to the control cells (Fig. 2, *E*, *F*, *I*, and *J*). Overexpression of CK1ε resulted in a decrease in the ratio of Bcl-xS/Bcl-xL mRNA and protein in HEK293T, but little effect was observed on Bcl-xS/Bcl-xL mRNA and protein ratio in CK1δ-overexpressing cells (Fig. 2, *G*, *H*, *K*, and *L*). Thus, CK1ε was selected for subsequent study. As expected, knockdown CK1ε could effectively increase the ratio of Bcl-xS/Bcl-xL mRNA and protein, resulted in an increased ratio of apoptotic cells in A549 cells (Fig. 3, *A*, *C*, *F*, and *H*). Treatment with SR3029 also enhanced the ratio of Bcl-xS/Bcl-xL (Fig. 3*D*). In addition, we noted that a kinase-dead mutant of CK1ε (CK1ε-K38R) had little effect on Bcl-xS to Bcl-xL mRNA and protein ratio in A549 cells comparing with WT CK1ε (Fig. 3, *B*, *E* and *G*), suggesting that the kinase activity is necessary to CK1ε-mediated Bcl-x alternative splicing.

### CK1ε interacts with SRSF10

**Figure 4:**
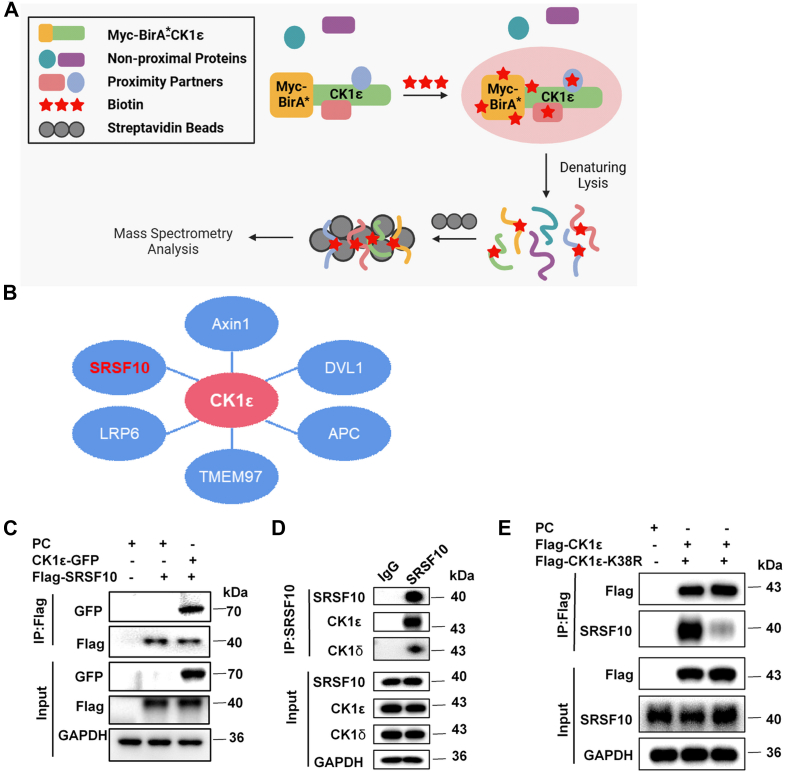
**SRSF10 interacts with CK1ε.***A*, scheme for proximity-dependent biotin identification (BioID) approach to identify the proteins interacting with CK1ε. *B*, the potential CK1ε-interacting proteins identified by mass spectrometry. *C*, HEK293T cells were cotransfected with expression plasmids for Flag-SRSF10 and the CK1ε-GFP as indicated, and subsequently immunoprecipitated with anti-Flag beads. *D*, A549 cell lysates were immunoprecipitated by IgG and anti-SRSF10 antibody, respectively, and endogenous interaction between CK1ε or CK1δ and SRSF10 was detected by Western blot. *E*, HEK293T cells were transfected with expression plasmids for Flag-CK1ε and Flag-CK1ε-K38R or control vector as indicated, and subsequently immunoprecipitated with anti-Flag beads. The endogenous interaction of SRSF10 and CK1ε or CK1ε-K38R was detected. CK1, casein kinase 1; HEK293T, human embryonic kidney 293T; SRSF, serine/arginine-rich splicing factor.

To further explore the molecular mechanism by which CK1ε regulates Bcl-x alternative splicing, a proximity-dependent biotin identification approach was used to identify CK1ε-interacting proteins. We generated stable A549 cells ectopically expressing CK1ε proteins tagged both Myc and BirA∗ (a mutant form of the biotin ligase BirA) under the control of a Tet-on promoter. CK1ε and proximal proteins were biotinylated. Streptavidin was used to purify the biotinylated protein, and the product was identified by mass spectrometry (MS) (Fig. 4*A*). We identified the RNA-binding protein SRSF10 as a potential interacting protein of CK1ε (Fig. 4*B*). Meanwhile, several previously known CK1ε interacting proteins including LRP6, Axin1, APC, DVL1, and TMEM97 were also identified, indicating the reliability of our assay (Fig. 4*B*). To further confirm the interaction between CK1ε and SRSF10, a coimmunoprecipitation (Co-IP) experiment was performed using HEK293T cells that were transiently transfected with GFP-tagged CK1ε and Flag-tagged SRSF10. The results showed that CK1ε could interact with SRSF10 (Fig. 4*C*).

We further assessed whether endogenous SRSF10 could interact with CK1ε or CK1δ in A549 cells. A Co-IP experiment was performed using anti-SRSF10 antibody. The results showed that endogenous SRSF10 could interact with CK1ε and CK1δ in A549 cells (Fig. 4*D*). However, much more CK1ε was pulled down by anti-SRSF10 antibody (Fig. 4*D*), indicating that the binding affinity of SRSF10 to CK1ε is higher than that to CK1δ. The higher binding affinity of CK1ε to SRSF10 may contribute to its selective activity in the regulation of SRSF10 alternative splicing. Consistently, knockdown of CK1δ or overexpression of CK1δ had little effect on the alternative splicing of Bcl-x (Fig. 2, *F*, *H*, *J*, and *L*).

HEK293T cells were transfected with the expression plasmids for CK1ε-Flag and the kinase-dead mutant CK1ε-K38R-Flag. The cell lysate was subjected to Western blot analysis using anti-Flag antibody. We confirmed the protein expression of CK1ε and CK1ε-K38R (Fig. 3*B*). We further test the effect of CK1ε and CK1ε-K38R on CK1ε-mediated signaling. CK1ε has been known to be a positive regulator of Wnt/β-catenin signaling. A Wnt-responsive SuperTOPFlash reporter assay was performed to test the effect of CK1ε on Wnt/β-catenin signaling. Our results showed that overexpression of CK1ε obviously increased the activity of SuperTOPFlash reporter, while the kinase-dead mutant CK1ε-K38R had much weaker effect on the activity of SuperTOPFlash reporter (Fig. S1), confirming the kinase-dead mutant of CK1ε is not active.

We next examined the effect of CK1ε kinase activity on the interaction between CK1ε and SRSF10. HEK293T cells were transfected with the expression plasmids for CK1ε-Flag and its kinase-dead mutant CK1ε-K38R-Flag. A Co-IP experiment was performed using anti-Flag antibody. The Co-IP results demonstrated that the kinase-dead mutation of CK1ε dramatically abolished the interaction of SRSF10 with CK1ε (Fig. 4*E*). Our results indicated that the interaction between CK1ε and SRSF10 is specific and functional, and the kinase activity of CK1ε is necessary to this interaction.

### SRSF10 and CK1ε are upregulated and may be implicated in Bcl-x splicing in lung cancer

The expression data were obtained from the TCGA-lung adenocarcinoma (LUAD) RNA-seq dataset in the TCGA database (https://portal.gdc.cancer.gov). The data were processed through the STAR pipeline and normalized using the transcripts per million method and subsequently log-transformed with a pseudocount of 1. The selected gene for analysis was SRSF10 (ENSG00000188529.15).

**Figure 5:**
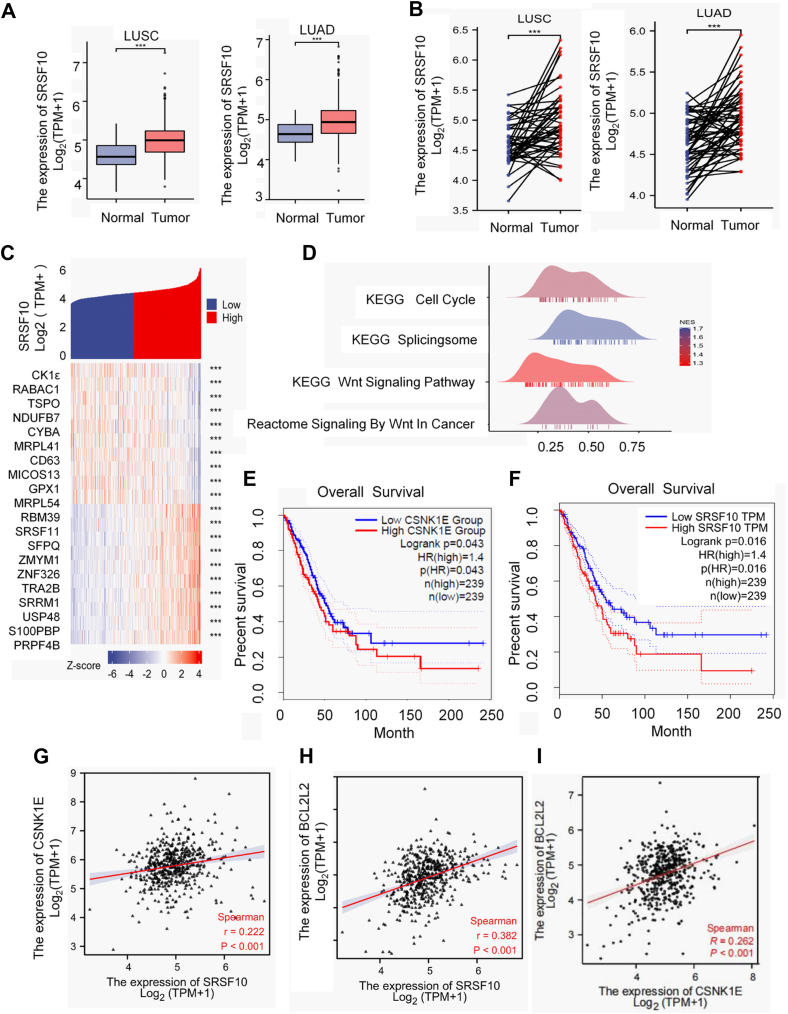
**The expression of SRSF10 is correlated with CK1ε expression in human lung cancer.***A*, the expression of SRSF10 in normal tissues and LUSC or LUAD from the TCGA database. Wilcoxon rank-sum test was used to compare the differences of SRSF10 expression between two groups. *B*, the variance in SRSF10 expression between adjacent normal tissues and paired samples of LUSC or LUAD from the TCGA database. Paired *t* test was used to compare the differences of SRSF10 expression between two groups. *C*, genes correlated with SRSF10 expression in TCGA lung cancer data were identified through single gene correlation analysis. Twenty representative genes were then selected for heat map visualization. *D*, the potential regulatory pathways of SRSF10 were investigated using gene set enrichment analysis (GSEA) based on these related genes. *E* and *F*, the survival curves were generated using the GEPIA database to assess and compare the impact of varying levels of CK1ε expression or SRSF10 expression on the overall survival outcomes of lung cancer patients. *G*–*I*, the correlation of mRNA expression between SRSF10, CSNK1E (CK1ε), and BCL2L1 (Bcl-x) was analyzed by TCGA database. CK1, casein kinase 1; HEK293T, human embryonic kidney 293T; SRSF, serine/arginine-rich splicing factor; LUAD, lung adenocarcinoma; LUSC, lung squamous cell carcinoma.

The expression data of SRSF10 in 539 samples of lung squamous cell carcinoma (LUSC) along with 72 paired adjacent noncancerous tissues, and 594 samples of LUAD with 59 paired adjacent noncancerous tissues obtained from the TCGA database. Wilcoxon rank-sum test was used to compare the differences of SRSF10 expression between normal and LUSC or LUAD tissues. Paired *t* test was used to compare the differences of SRSF10 expression between adjacent normal tissues and paired samples of LUSC or LUAD tissues. Our results showed that SRSF10 expression was higher in both LUSC and LUAD tissues than that in normal tissues or adjacent noncancerous tissues (Fig. 5, *A* and *B*).

Gene set enrichment analysis indicated the potential role of SRSF10 in spliceosomes, the Wnt signaling pathway, and the cell cycle process (Fig. 5, *C* and *D*). The correlation analysis conducted using the GEPIA 2.0 database revealed that high expression of CK1ε in LUAD patients was associated with decreased overall survival, with a *p* value of 0.043 and a hazard ratio (HR) of 1.4 (Fig. 5*D*). Furthermore, high expression of SRSF10 was also associated with lower overall survival, with a *p* value of 0.016 and a HR of 1.4 (Fig. 5*E*). These results suggest that increased CK1ε and SRSF10 expression might have significant implications for the clinical outcome of LUAD. Additionally, the correlation analysis showed that SRSF10, expression of CK1ε, and BCL2L1 (Bcl-x) were all positively correlated with each other (Fig. 5, *F*–*H*). Taken together, these results imply that SRSF10 and CK1ε may play an important role in pathogenesis of lung cancer, and SRSF10 may be involved in the expression of Bcl-x splicing.

### CK1ε regulates Bcl-x alternative splicing through SRSF10 in lung cancer cells

**Figure 6:**
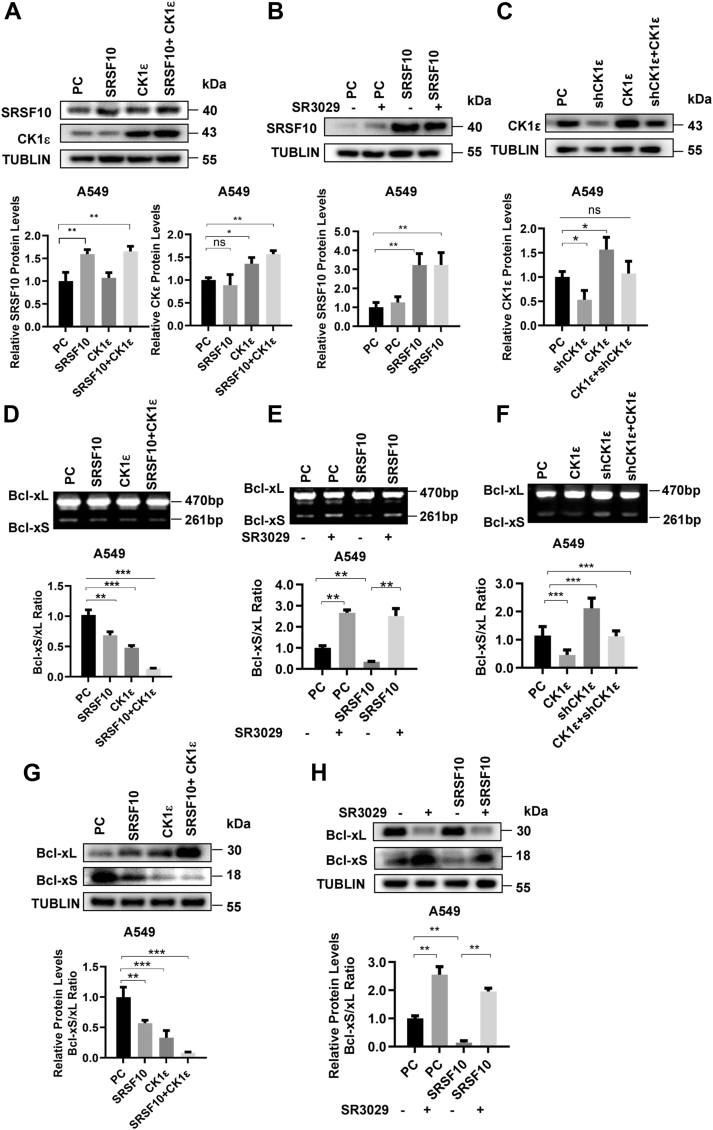
**SRSF10 regulates the alternative splicing of Bcl-x and CK1ε is involved in SRSF10-mediated Bcl-x splicing.***A*–*C*, the protein expression of CK1ε and SRSF10 in A549 cells with knockdown or overexpression of CK1ε and SRSF10 was detected by Western blot. The protein quantitative results of the grayscale were shown below. *D*, A549 cells were transfected with SRSF10 expression vector with or without CK1ε expression plasmid. The alternative splicing of Bcl-x was detected by RT-PCR. The quantitative results of the grayscale ratio of Bcl-xS to Bcl-xL are shown below. *E*, A549 cells were transfected with SRSF10 expression vector in the presence or absence of SR3029. The alternative splicing of Bcl-x was detected by RT-PCR. A representative gel was demonstrated, and the quantitative results of the grayscale ratio of Bcl-xS to Bcl-xL are shown below. *F*, CK1ε rescued the CK1ε knockdown-induced alternative splicing changes of Bcl-x in A549 cells. The alternative splicing of Bcl-x was detected by RT-PCR. The quantitative results of the grayscale ratio of Bcl-xS to Bcl-xL are shown below. *G* and *H*, the protein expression levels of Bcl-xL and Bcl-xS in A549 cells with overexpression of CK1ε and SRSF10 in the presence or absence of SR3029 were analyzed by Western blot. The protein quantitative results of the grayscale ratio of Bcl-xS to Bcl-xL are shown below. CK1, casein kinase 1; HEK293T, human embryonic kidney 293T; SRSF, serine/arginine-rich splicing factor.

To assess the role SRSF10 in CK1ε-mediated Bcl-x splicing in lung cancer, A549 cells were transfected with SRSF10 or CK1ε expression plasmids alone or combined together. The protein expression of SRSF10 and CK1ε was detected in A549 cells expressing SRSF10 or/and CK1ε (Fig. 6*A*). Either SRSF10 or CK1ε reduced the ratio of Bcl-xS/Bcl-xL mRNA and protein (Fig. 6, *D* and *G*). Simultaneous expression of SRSF10 and CK1ε exhibited synergistic effect on Bcl-xS/Bcl-xL ratio mRNA and protein (Fig. 6, *A*, *D*, and *G*). SR3029 treatment abolished the effect of SRSF10 on the ratio of Bcl-xS/Bcl-xL mRNA and protein (Fig. 6, *B*, *E*, and *H*).

To exclude off-target effect of shRNA, a rescue experiment was performed by re-expressing CK1ε in A549 cells with CK1ε knockdown. The expression of CK1ε was confirmed in A549 cells with re-expressing CK1ε by Western blot (Fig. 6*C*). The effect of CK1ε knockdown on Bcl-x’s alternative splicing was reversed upon reintroduction of CK1ε in A549 cells (Fig. 6*F*).

We next evaluated the effect of CK1ε/SRSF10 axis on Bcl-x alternative splicing in lung cancer cells, the expression of SRSF10 was knocked down. We also overexpressed CK1ε in A549 cells (Fig. S2*A*). The RT-PCR results showed that silencing SRSF10 enhanced the ratio of Bcl-xS/Bcl-xL mRNA, while CK1ε overexpression had opposite effect (Fig. S2*B*). The overexpression of CK1ε also abrogated the impact of SRSF10 knockdown on the ratio of Bcl-xS/Bcl-xL mRNA (Fig. S2*B*). These results illustrated that CK1ε mediated Bcl-x alternative splicing through SRSF10 in lung cancer cells.

**Figure 7:**
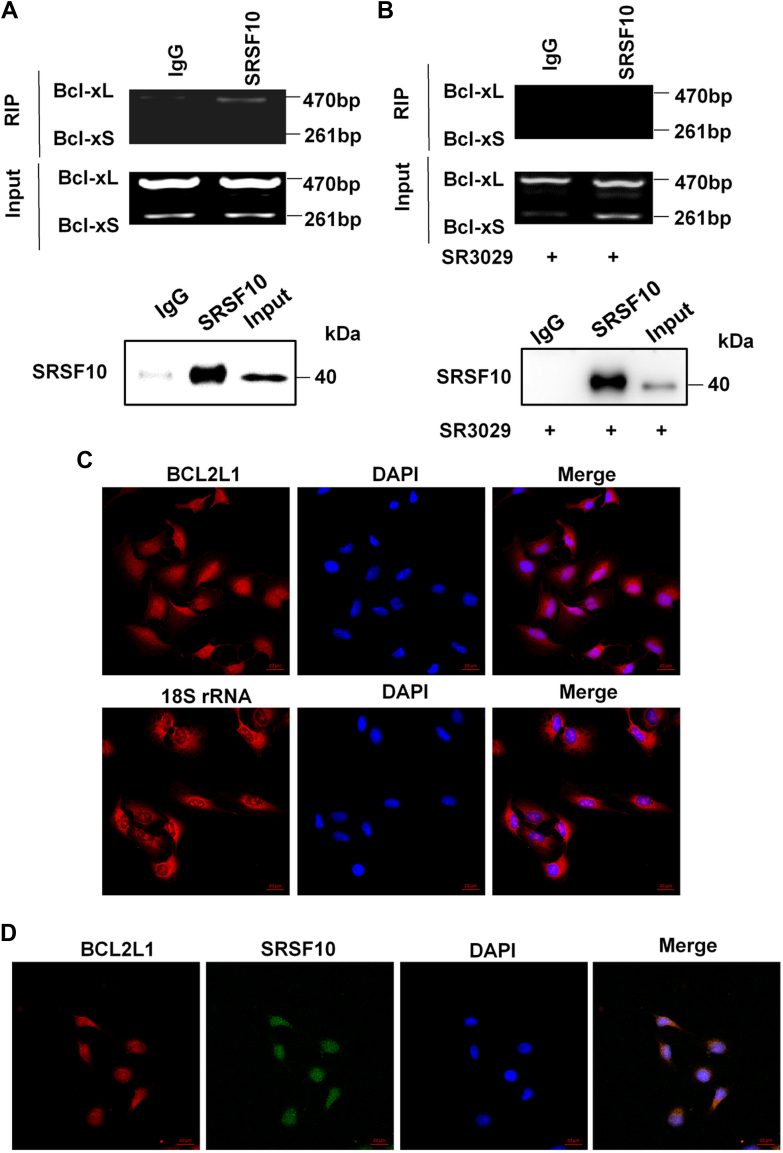
**SRSF10 interacts with Bcl-x pre-mRNA.***A*, binding of SRSF10 protein with Bcl-x pre-mRNA was examined by RNA-immunoprecipitation assay in A549 cells exogenously overexpressing SRSF10 using an anti-SRSF10 antibody or control IgG. *B*, A549 cells were treated with 120 nM SR3029 for 24 h, and the effect of SR3029 on the binding of SRSF10 to Bcl-x pre-mRNA was detected by RNA-immunoprecipitation assay. *C*, confocal FISH images showing localization of BCL2L1 (18S, probe for 18S rRNA). *D*, the combination of FISH and IFS methods showed the colocalization of the SRSF10 protein and BCL2L1 mRNA in A549 cells. The scale bar represents 20 μm. IFS, immunofluorescent staining.

To determine whether SRSF10 is able to bind to Bcl-x mRNA, an RNA immunoprecipitation assay was performed. The results showed that SRSF10 could selectively bind to Bcl-xL splicing isoform, while SR3029 significantly inhibited the binding of SRSF10 to Bcl-xL mRNA (Fig. 7, *A* and *B*). To further study the underlying mechanism by which SRSF10 mediates Bcl-x splicing, we examined the cellular distribution of BCL2L1 mRNA by confocal microscopy using FISH assay. In A549 cells, BCL2L1 was found to be located in both the nucleus and cytoplasm (Fig. 7*C*). The combination of FISH and immunofluorescent staining methods showed the colocalization of the SRSF10 protein and BCL2L1 mRNA in A549 cells (Fig. 7*G*). We did not observe the colocalization of SRSF10 protein and BCL2L1 mRNA in A549 cells with SRSF10 knockdown (Fig. S3). Collectively, these results indicate that CK1ε–SRSF10 axis regulates the alternative splicing of Bcl-x, and phosphorylation modification of SRSF10 by CK1ε may influence its binding with Bcl-x mRNA.

### CK1ε promotes the alternative splicing of Bcl-x by phosphorylation of SRSF10

**Figure 8:**
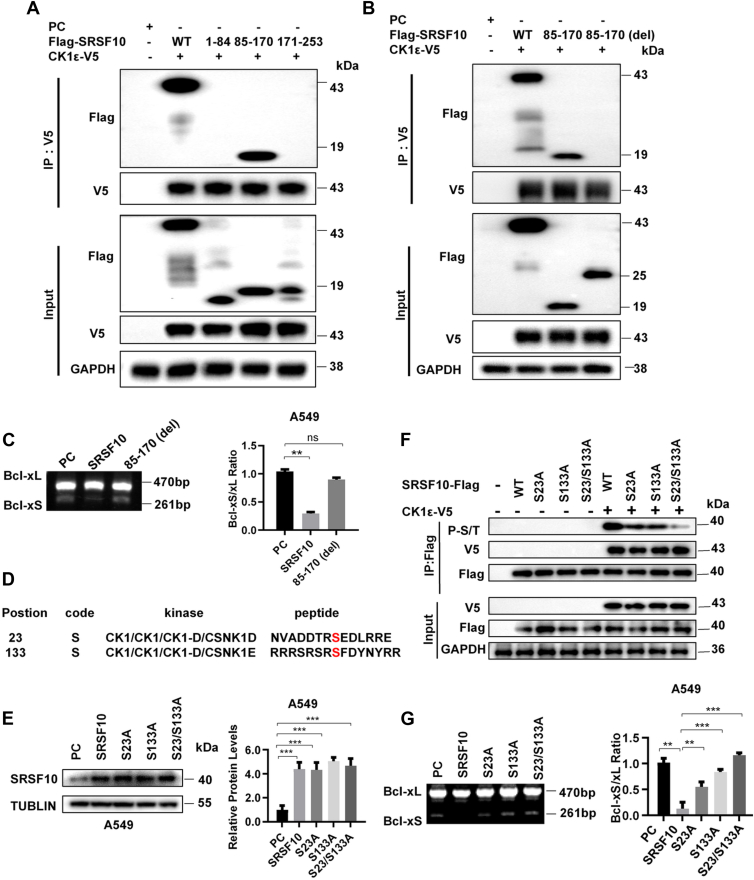
**CK1ε phosphorylates SRSF10 at Ser23/133 to modulate the alternative splicing of Bcl-x.***A*, the truncation mutants of SRSF10, 1 to 84, 85 to 170, and 171 to 253 were generated. HEK293T cells were cotransfected with expression plasmids for Flag-SRSF10 (WT), 1 to 84, 85 to 170, 171 to 253, and the CK1ε-V5 as indicated, and subsequently immunoprecipitated with anti-V5 beads. *B*, HEK293T cells were cotransfected with expression plasmids for Flag-SRSF10 (WT), its truncation mutants 85 to 170, 85 to 170 (del), and the CK1ε-V5 as indicated, and subsequently immunoprecipitated with anti-V5 beads. *C*, the effect of Flag-SRSF10 (WT) and its truncation mutants 85 to 170, 85 to 170 (del) on alternative splicing changes of Bcl-x in A549 cells. The alternative splicing of Bcl-x was detected by RT-PCR. The quantitative results of the grayscale ratio of Bcl-xS to Bcl-xL are shown on the *right*. *D*, two CK1 phosphorylation sites in the SRSF10 protein sequence were predicted by GPS based on the consensus CK1 phosphorylation motif of pS/pT-X1-2-S/T. *E*, the protein expression of SRSF10 in A549 cells with overexpression of SRSF10 and its mutants was detected by Western blot. The protein quantitative results of the grayscale were shown on the *right*. *F*, CK1ε-V5 expression vector was cotransfected with expression plasmids for SRSF10-S23A-Flag, SRSF10-S133A-Flag, or SRSF10-S23/S133A-Flag into HEK293T cells, as indicated. The interaction between CK1ε and WT or mutated SRSF10 was detected by coimmunoprecipitation assay. The expression of serine-phosphorylated SRSF10 was detected by an anti-phospho-serine/threonine antibody. *G*, A549 cells were transfected with expression vectors for SRSF10-Flag, SRSF10-S23A-Flag, SRSF10-S133A-Flag, or SRSF10-S23/S133A-Flag, as indicated. The effect of WT SRSF10 and SRSF10 mutants on the alternative splicing of Bcl-x was analyzed by RT-PCR. The quantitative results of Bcl-xS to Bcl-xL ratio are shown on the *right*. CK1, casein kinase 1; HEK293T, human embryonic kidney 293T; SRSF, serine/arginine-rich splicing factor.

To determine the domain of interaction of SRSF10 with CK1ε, we generated several truncation mutants of SRSF10, 1 to 84, 85 to 170, 171 to 253, which contains the 1 to 84, 85 to 170, and 171 to 253 amino acid residues, respectively. The results from the Co-IP experiments showed that CK1ε interacted with the 85 to 170 mutant of SRSF10 (Fig. 8*A*). In contrast, when the 85 to 170 region of SRSF10 was deleted, the truncated protein no longer interacted with CK1ε (Fig. 8*B*). Compared with WT SRSF10, its truncated protein 85 to 170 had no effect on the alternative splicing of Bcl-x (Fig. 8*C*). These results indicated that the 85 to 170 region of SRSF10 mediates CK1ε interaction.

CK1ε is a serine/threonine protein kinase that may modulate Bcl-x alternative splicing by phosphorylation of SRSF10. The sequence analysis revealed that SRSF10 has two consensus CK1ε target phosphorylation motif at amino acids S23 and S133 (Fig. 8*D*). We generated S23A, S133A, and S23A/S133A SRSF10 mutants in constructs expressing Flag-tagged SRSF10. HEK293T cells were transfected with WT SRSF10, SRSF10-S23A-Flag, SRSF10-S133A-Flag, and SRSF10-S23A/S133A-Flag alone or along with CK1ε-V5 expression vector. Western blot confirmed the protein expression of SRSF10 and its mutants (S23A, S133A, and S23A/S133A) (Fig. 8*E*). The results from the Co-IP experiment showed that the SRSF10 mutations at S23 and S133 had little effect on the interaction between CK1ε and SRSF10. Importantly, the levels of phosphorylated SRSF10 by CK1ε were markedly decreased upon single point mutations at S23 or S133. A double point mutation at S23/133 completely abrogated CK1ε-mediated phosphorylation of SRSF10 (Fig. 8*F*). These results indicated that CK1ε may phosphorylate SRSF10 at S23 and S133, which required for CK1ε–SRSF10 axis-mediated Bcl-x splicing. Consistently, the point mutants of SRSF10 at S23 or S133 have less effect on Bcl-xS/Bcl-xL ratio compared with WT SRSF10, whereas the expression of a double point mutant at S23/133 had no effect on Bcl-x splicing (Fig. 8G). In addition, MS was used to identify CK1ε-mediated phosphorylation sites in SRSF10. HEK293T cells expressing Flag-tagged SRSF10 and V5-tagged CK1ε were lysed and subjected to anti-Flag immunoprecipitation. The immunoprecipitated complexes were washed extensively, resolved by SDS-PAGE, and the complex band was excised for in-gel tryptic digestion. MS analysis demonstrated a significantly increased phosphorylation of S23 and S133 sites in SRSF10 in the presence of CK1ε (Fig. S4).

The ADP-Glo kinase assay was used to determine if CK1ε may directly phosphorylate SRSF10 at S23 and S133. The ADP-Glo kinase assay was performed using the ADP-Glo kinase assay kit obtained from Promega. After kinase buffer was added to a 384-well plate, the synthetic peptide of SRSF10, and its mutated peptides (S23A, S133A, and S23/133A) were incubated with the purified CK1ε enzyme. Then, the ADP-Glo reagent and the kinase detection reagent were added and incubated for 30 min, respectively. The ATP was measured by detecting the luminescence signal using a microplate reader. The magnitude of the luminescence signal is directly proportional to the CK1ε-mediated phosphorylation of SRSF10 peptides. As shown in Fig. S5, addition of purified CK1ε enzyme to a kinase reaction mixture containing WT SRSF10 peptide resulted in a strong luminescence signal in a dose-dependent manner, while the weaker luminescence signal was observed in the presence of S23A and S133A mutated peptides. The weakest luminescence signal was detected in the presence of the double mutated peptide (S23/S133A) (Fig. S5, *A* and *B*). Similarly, we observed an enhanced luminescence signal in the presence of increasing concentrations of different peptides (Fig. S5, *A* and *B*). These results indicated that CK1ε could directly phosphorylate SRSF10 at S23/133, which required for CK1ε–SRSF10 axis-mediated alternative splicing of Bcl-x.

### SR3029 potentiates the effect of SRSF10 knockdown on apoptosis, viability, proliferation, and clonogenic formation in lung cancer cells

**Figure 9:**
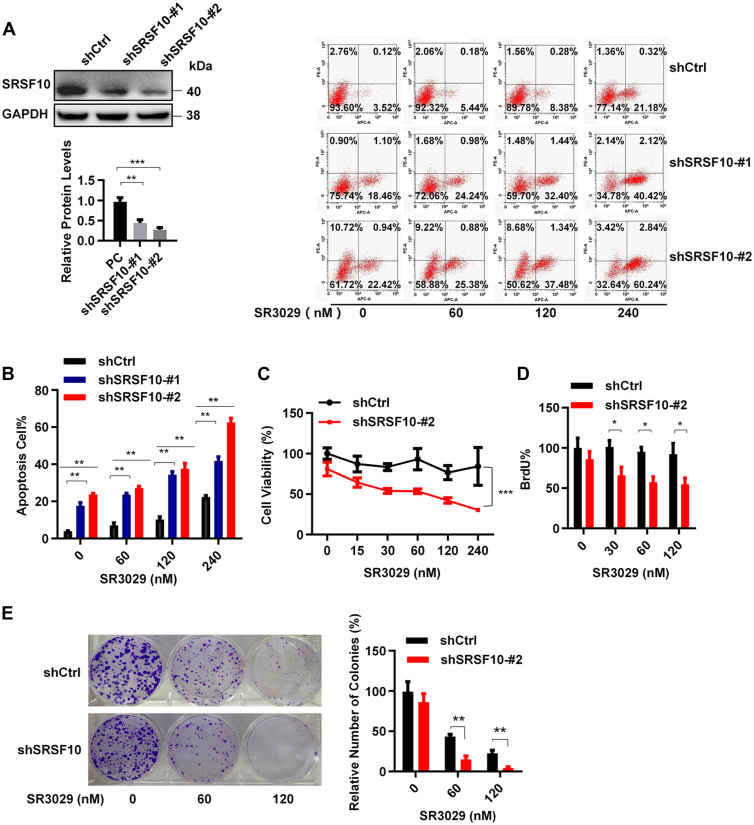
**SR3029 potentiates the effect of SRSF10 knockdown on apoptosis, viability, proliferation, and clonogenic formation in A549 cells.***A*, A549 cells were infected with the indicated lentivirus. The expression of SRSF10 was detected by Western blot. *B*, treatment with SR3029 further enhanced SRSF10-mediated apoptosis. The SRSF10-knockdown A549 cells and their parental cells were treated with increasing concentrations of SR3029 as indicated. Cell apoptosis was detected by flow cytometer. *C*, the SRSF10-knockdown A549 cells and their parental cells were inoculated in 96-well plates and treated with different concentrations of SR3029 for 48 h. The effect of SR3029 treatment on cell viability was detected by 3-(4,5-dimethyl-2-thiazolyl)-2,5-diphenyl-2-H-tetrazolium bromide. *D*, the SRSF10-knockdown A549 cells and their parental cells were inoculated in 96-well plates and treated with different concentrations of SR3029 for 48 h. The effect of SR3029 treatment on cell proliferation was examined by BrdU incorporation assay. *E*, the SRSF10-knockdown A549 cells and their parental cells (500 per well) were inoculated in 6-well plate, treated with different concentrations of SR3029 for 14 days. The clonal formation capacity of the cells was examined by 1% crystal violet staining. *Right panel:* graphical representation of quantitative data showed the relative number of colony formation (n = 6). The scale bar represents 200 μm. SRSF, serine/arginine-rich splicing factor.

SRSF10 has been found to be highly expressed in lung carcinoids (32). To evaluate the role of SRSF10 in the oncogenesis of lung cancer, lentivirus-mediated shRNAs were used to knock down the expression of SRSF10 in lung cancer A549 cells. The knockdown efficiency was confirmed by Western blot analysis (Fig. 9*A*). Our results showed that the knockdown efficiency of shSRSF10#2 was better and shSRSF10#2 was used in subsequent experiments (Fig. 9*A*). Depletion of SRSF10 significantly enhanced apoptosis in A549 cells, while CK1ε inhibitor SR3029 further potentiated the effect of SRSF10 knockdown on apoptosis in lung cancer cells (Fig. 9*B*). Knockdown of SRSF10 also slightly reduced the viability, proliferation, and colony formation (Fig. 9, *C*–*E*). Moreover, silencing SRSF10 increased the sensitivity of A549 cells to SR3029. Treatment with SR3029 and knockdown of SRSF10 exerted a stronger inhibitory effect on the viability, proliferation, and colony formation in lung cancer cells (Fig. 9, *C*–*E*). These results suggest that CK1ε–SRSF10 axis may be involved in pathogenesis of lung cancer.

### Knockdown of SRSF10 inhibits lung tumor growth *in vivo*, and SR3029 potentiates the effect of SRSF10 deficiency

**Figure 10:**
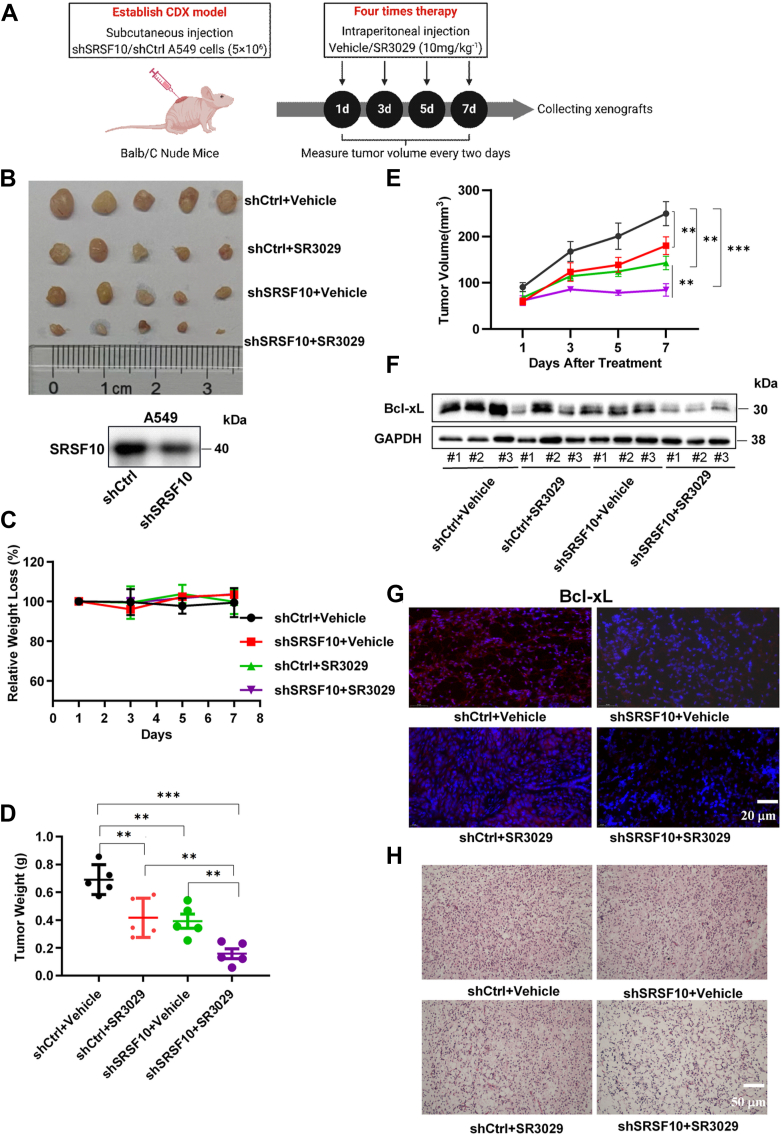
**SR3029 enhances the inhibitory effect of SRSF10 deficiency on lung tumor growth *in vivo*.***A*, the SRSF10-knockdown A549 cells and their parental cells were subcutaneously (s.c.) implanted into the right back of 7-week-old male Balb/c nude mice to generate a cell line-derived xenograft (CDX) tumor model. The SRSF10-knockdown effect in A549 cells were verified by Western blot. When the tumors reached about 50 mm^3^, mice were randomly divided into two groups and intraperitoneally (i.p.) injected with the vehicle (10% dimethyl sulfoxide/40% PEG-300/5% Tween-80/45% saline) or SR3029 (10 mg/kg BW) every 2 days. Tumor sizes were measured with a caliper and tumor volumes were calculated using the formula: 0.528 × length × width^2^. At 7 days after SR3029 treatment, mice were sacrificed, and tumors were collected and photographed. *B*, images of tumors from each experimental group. *C*, the body weight loss in each experimental group. *D*, mean tumor weight in each experimental group. *E*, mean tumor volume in each experimental group. *F*, immunoblot analysis of Bcl-xL in each experimental group. *G*, immunofluorescence staining of Bcl-xL in each experimental group. Nuclei were counterstained with DAPI. *H*, representative images of H&E staining in each experimental group, the scale bars represent 50 μm. DAPI, 4,6-diamidino-2-phenylindole.

To explore the effects of CK1ε–SRSF10 axis on tumor growth *in vivo*, SRSF10-knockdown A549 cells and their parental counterparts were subcutaneously injected into Balb/c mice to generate a xenograft tumor model (Fig. 10*A*). When the tumors reached about 50 mm^3^, mice were randomly divided into two groups and intraperitoneally injected with the vehicle or SR3029 (10 mg/kg body weight) every 2 days. Since tumor volume was obviously decreased after treatment with SR3029 for 7 days compared with the control mice, mice were sacrificed 7 days after the treatment. We observed reduced tumor growth in mice bearing SRSF10 deficiency tumors compared with control mice (Fig. 10*B*). Mice inoculated with SRSF10-knockdown cells and administered SR3029 had the slowest tumor growth, but had no effect on body weight (Fig. 10, *C*–*E*), concomitant with decreased protein level of Bcl-xL (Fig. 10, *F* and *G*). Histological analyses revealed that either SRSF10 deficiency or SR3029 treatment reduced tumor cell density, and the combination of SRSF10 deficiency and SR3029 treatment exerted strongest inhibitory effect on tumor cell density (Fig. 10*H*). Collectively, these results illustrated that SRSF10 knockdown combined with SR3029 administration synergistically inhibited lung tumor growth through downregulating Bcl-xL expression.

In addition, we compared the effect of CK1ε or SRSF10 silencing with the standard therapeutic agent cisplatin on lung cancer in mice. The administration of cisplatin and knockdown of SRSF10 or CK1ε effectively suppressed the growth of lung cancer without changes in body weight, while silencing SRSF10 exerted strongest inhibitory effect on tumor growth (Fig. S6, *A*–*D*). Moreover, the tumors treated with cisplatin or bearing deficiency of CK1ε and SRSF10 had decreased protein level of Bcl-xL and reduced tumor cell density (Fig. S6, *E* and *G*). These results indicate that CK1ε–SRSF10 axis plays a critical role in regulating the growth of lung cancer.

## Discussion

**Figure 11:**
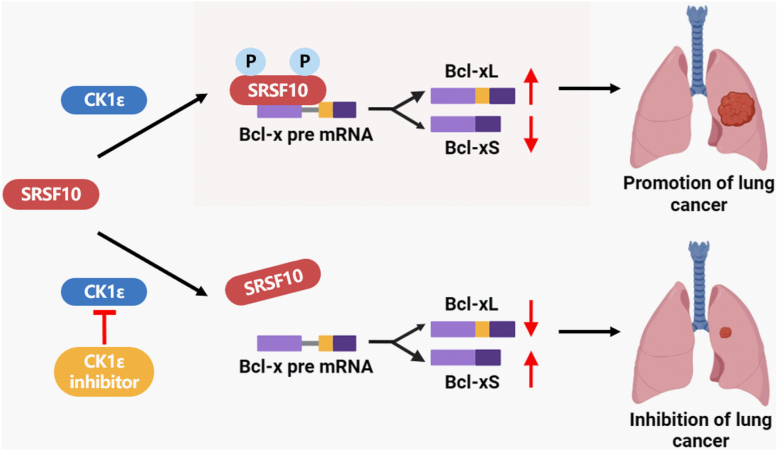
**A novel mechanism by which the CK1ε–SRSF10 axis regulates Bcl-x alternative splicing and tumorigenesis in lung cancer.** CK1, casein kinase 1; SRSF, serine/arginine-rich splicing factor.

Deregulation of CK1ε has been found to be implicated in multiple diseases, including cancers (33, 34, 35). In LUAD, the long noncoding RNA LINC00673 promoted invasiveness, migration, and metastasis of cancer cells (36). Mechanistically, LINC00673-v4 could augment the association of DDX3 with CK1ε, resulting in the phosphorylation of disheveled and activation of Wnt/β-catenin signaling (36). In colorectal cancer, CK1δ/ε-induced amino-terminal enhancer of split degradation resulted in the activation of the Wnt and Notch signaling cascades, thereby increasing the proliferation, migration, invasion, and sphere formation of colon cancer cells, promoting tumor growth and metastasis (30). Ning *et al.* reported that CK1δ/ε could increase β-catenin–mediated transcriptional activity through regulating βcatenin acetylation at K49 (37). CK1δ/ε enhanced the interaction between β-catenin and Tip60, and recruited Tip60 to β-catenin complex, thereby leading to the acetylation of β-catenin at K49. Blockade of CK1δ/ε or/and Tip60 downregulated β-catenin acetylation and the transcription of Wnt target genes, resulting in growth inhibition of colon cancer cells (37). Moreover, CK1ε exerted its oncogenic role in occurrence and progression of colorectal cancer by regulating the stability of Axin1 (31). In this study, we found that CK1ε could mediate the alternative splicing of Bcl-x *via* phosphorylation of SRSF10. Interestingly, we noted that the binding between SRSF10 and CK1ε alone was not enough for SRSF10’s activity, and CK1ε-mediated phosphorylation of SRSF10 at S23/133 was critical for SRSF10’s activity in Bcl-x’s alternative splicing. This study elucidates a novel mechanism by which the CK1ε–SRSF10 axis regulates Bcl-x alternative splicing and tumorigenesis in lung cancer (Fig. 11).

It has been noted that SRSF10 exhibited its tumorigenic effect *via* modulating alternative splicing of Bcl-x and pyruvate kinase M (38). As a transcription factor, early growth response 1 (EGR1) upregulated the expression level of SRSF10 by recruiting ten-eleven translocation protein 1 at the demethylated EGR1 binding site, while inhibition of the ERK1/2 pathway decreased the expression of EGR1 and SRSF10 (38). DNA damage induced by oxaliplatin could enhance the dephosphorylation of SRSF10 and induce the generation of proapoptotic Bcl-xS isoform (18). Splicing factors hnRNP A1/A2 and Sam68 also collaborated with SRSF10 to regulate the alternative splicing response to oxaliplatin (39). SRSF10 has been demonstrated to be dephosphorylated by the phosphatase PP1 in response to heat shock (40, 41). However, it remain unclear how SRSF10 phosphorylation modulates its activity in the process of alternative RNA splicing. Here, we found that CK1ε could interact with and phosphorylate SRSF10 at S23/133, which may affect its binding with Bcl-xL mRNA. Future studies are needed to address how phosphorylated SRSF10 affects its interaction with the Bcl-x pre-mRNA.

Previous studies reported that Bcl-x splicing was dysregulated in a large percentage of human non–small cell lung cancer tumors (42). Shapiro *et al.* reported that melanoma differentiation–associated gene 7/IL-24 could alter the alternative splicing of Bcl-x through the SRC/PKCδ signaling axis in non–small cell lung cancer cells (43). The splicing factor SAP155 was found to bind to ceramide-responsive RNA *cis-*element 1, which was required for ceramide to induce the activation of the Bcl-x(S) 5′ splice site (44). Tumor suppressor RBM10 could also enhance selection of an internal 5′-splice site in Bcl-x pre-mRNA. Its deficiency increased the expression of antiapoptotic isoform Bcl-xL, leading to tumor progression and worse clinical outcomes in EGFR-mutant lung cancer (45). A recent study showed that tumor cell–derived GM-CSF could increase the expression of the antiapoptotic isoform Bcl-xL and enhance neutrophil survival *via* JAK/STAT signaling in LUAD (46). Our studies showed that knockdown of SRSF10 could enhance apoptosis and inhibit the viability, proliferation, and clonogenic formation in lung cancer cells, while CK1 inhibitor SR3029 further potentiated the effect of silencing SRSF10 on lung cancer cells. In A549 xenograft tumor model, SRSF10 knockdown combined with SR3029 administration synergistically inhibited tumor growth, accompanied with decreased protein level of Bcl-xL. Compared with cisplatin, a standard chemotherapeutic agent for lung cancer, silencing SRSF10, and CK1ε exhibited potent inhibitory effects on the growth of lung cancer. These results indicated that the CK1ε/SRSF10-mediated Bcl-x splicing is involved in pathogenesis of lung cancer, which may be a potential target for lung cancer treatment.

## Experimental procedures

### Cell culture and treatment

Human lung cancer A549 cells and (HEK293T) cells were acquired from American Type Culture Collection and grown in Dulbecco's modified Eagle's medium containing 10% fetal bovine serum and 1% penicillin-streptomycin (Thermo Fisher Scientific). The SRSF10-KO A549 cell line was generated using standard protocol. All cell cultures were maintained in a humidified incubator at 37 °C with 5% CO_2_.

### Lentiviral production and infection

The SRSF10 shRNA oligos were cloned into pLKO.1-GFP vector. The resulting construct was validated by sequencing. HEK293T cells were transfected with a pLKO.1-GFP-SRSF10 viral plasmid, an envelope plasmid pMD2.G and a packaging plasmid psPAX2 using Lipofectamine 2000 regent according to manufacturer’s instruction. After transfection, the virus-containing supernatants were collected. Virus was immediately added to A549 cells. After a week of infection, the GFP-positive cells were sorted by FACS AriaIII, and the knockdown of SRSF10 was verified by Western blotting. The sequences of SRSF10 shRNAs were as follows: Sh SRSF10-1#: CCGGACAACTATAGAAGATCGTATACTCGAGTATACGATCTTCTATAGTTGTTTTTTG; ShSRSF10-2#: CCGG CGTTCTAGAAGCCGAAGTTATCTCGAGATAACTTCGGCTTCTAGAACGTTTTTG.

### Semiquantitative RT-PCR analysis

Total RNA was isolated by RNAiso Plus (TaKaRa) and reverse-transcribed into complementary DNA (cDNA) using the PrimeScript RT reagent kit (TaKaRa) according to the manufacturer’s instructions. To analyze alternative splicing of exon 2 in the Bcl-x gene, 5′ primer to Bcl-x (5′-GAGGCAGGCGACGAGTTTGAA-3′), and 3′ primer (5′-TGGGAGGGTAGAGTGGATGGT-3′) were used for PCR amplification (30 cycles, 94 °C, 30 s; 56 °C, 30 s; 72 °C, 1 min) with 2xEasy Taq superMix (Transgen Biotech). The lengths of splicing variants of Bcl-xL and Bcl-xS are 460 bp and 271 bp, respectively. PCR products were separated and analyzed on agarose gels, with the bands of the Bcl-xL and Bcl-xS splicing variants being confirmed by DNA sequencing.

### RNA immunoprecipitation assay

RNA-binding protein immunoprecipitation (RIP) analysis was conducted using the Magna RIP kit (Millipore) and SRSF10 antibody (Santacruz, sc-101132) following the manufacturer’s protocol. A549 cells treated with SR3029 and vehicle control were lysed in RIP lysis buffer. The nuclear substances were enriched with a SRSF10 antibody and IgG control, respectively, and the beads of proteinA/G were added on the second day and incubated at 4 °C for 1 h. After washing the RNA–protein complex for five times, protease K and DNAase were added to remove impurities. Total RNAs from cell lysates and immunoprecipitated fractions were used for PCR analyses.

## Combination of FISH and immunofluorescent staining

Combining FISH and immunofluorescent staining presents a powerful method for colocalizing mRNA and proteins in different tissues (47, 48).

The probe sets for FISH were purchased from a commercial source (RiboBio). In order to achieve a sufficient signal-to-background ratio, multiple probes were targeted along each individual long non-coding RNA sequence. A set of 15 to 20 probes that cover the entire length of the RNA molecule provided an optimal signal strength and each probes carrying multiple fluorophores. The pooled FISH probes were resuspended to a final concentration of 20 μM in RNase-free storage buffer and protected from light at −20 °C.

*In situ* hybridization was performed with a FISH Kit (RiboBio). Cells were briefly rinsed in PBS and fixed in 4% formaldehyde for 30 min. Then, the cells were permeabilized in PBS containing 1% Triton X-100 at room temperature for 30 min, washed with PBS three times, and prehybridizated at 37 °C for 30 min before hybridization. Then, either anti-BCL2L1 or anti-18S oligodeoxynucleotide probe were used in the hybridization solution at 37 °C overnight in the dark.

Immunofluorescence labeling was followed by FISH. The cells were washed with PBS three times, and then blocked with 5% normal goat serum for 30 min. Thereafter, cells were incubated with primary antibody against SRSF10 (Santa cruz) at 4 °C overnight, and then incubated with the specified secondary antibodies for 1 h. Nuclei were counterstained with 4,6-diamidino-2-phenylindole, and the coverslips were mounted on a glass slide. Images were captured with a LSM 5 EXCITER confocal imaging system (Carl Zeiss).

### Cell apoptosis assay

Cell apoptosis assay was performed using BioLegend's APC Annexin V Apoptosis Detection Kit (BioLegend). Cells (1–10 × 10^5^) were collected and resuspended in binding buffer. Then cells were incubated with Annexin V-PE and 7-aminoactinomycin D fluorescein solutions (Multi Sciences) according to the manufacturer’s protocol. Images of cell apoptosis were acquired by flow cytometer. FlowJo v10.0.8 (TreeStar Inc; http://www.flowjo.com/) was used for subsequent analysis.

### Cell viability assay

SRSF10-knockdown A549 cells and control cells were seeded in 96-well plate (3000 cells per well) and incubated in humidified incubator at 37 °C with 5% CO_2_. Cells were treated with different concentrations of SR3029, 10 μl of 5 mg/ml 3-(4,5-dimethyl-2-thiazolyl)-2,5-diphenyl-2-H-tetrazolium bromide (Diamond, 298-93-1) solution was added to the cells at different time points. Then, 100 μl dimethyl sulfoxide was added and absorbance was measured at 570 nm.

### Colony formation assay

SRSF10-knockdown A549 cells and control cells were seeded in 6-well plate (500 cells per well) and incubated in humidified incubator at 37 °C with 5% CO_2_ for 2 weeks. The cells were treated with different concentrations of SR2029 every 3 days. The clones were washed with PBS, fixed with 4% paraformaldehyde, and dyed with 1% crystal violet solution.

### Immunoblot analysis

Cells were initially congregated and lysed in lysis buffer consisting of 20 mM Tris–HCl (pH 7.4), 150 mM NaCl, 2.5 mM sodium pyrophosphate, 1 mM EDTA, 1 mM EGTA, 1 mM PMSF, 1% Triton X-100, 1 mM β-glycerol phosphate, 1 mM sodium orthovanadate, and 2 μg/ml leupeptin together with the protease inhibitor PMSF, following with sonication. The bicinchoninic acid protein assay kit (Cell Signaling Technology, Inc.) was then applied to ascertain the concentration of proteins. Equal amounts of proteins were then separated by SDS-PAGE in an 8% SDS-polyacrylamide gel followed by transferring to polyvinylidene difluoride membranes (Millipore). Western blotting was operated at 4 °C overnight with primary antibodies as follows: anti-Bcl-xL (Cell Signaling Technology, 2764S), anti-Bcl-xS (Invitrogen, PA5-32203) and anti-tublin (Proteintech, 60031-1-Ig), and anti-β-actin (TransGen Biotech, HC201-01). Blocking with 5% nonfat powdered milk (Sangon Biotech) at room temperature for 1 h, the polyvinylidene difluoride membranes were then incubated for another hour at room temperature with relevant secondary antibody horseradish peroxidase-conjugated goat anti-mouse (Thermo Fisher Scientific, Cat#A16066) or anti-rabbit (Thermo Fisher Scientific, Cat#A16096) IgG. The blots were eventually visualized by Tanon5200 Chemiluminescent Imaging System (Tanon) or X-ray film (Kodak) after incubation with ECL Plus Western Blotting Substrate (Thermo Fisher Scientific). The outcomes of fluorescence reaction were then analyzed by the ImageJ 1.8.0 Analysis Software (National Institutes of Health; https://imagej.nih.gov/ij/).

### Protein immunoprecipitation assay

Cells were treated with RIP analysis lysis buffer (50 mM Tris–HCl, pH7.4, 150 mM NaCl, 1% NP-40, 0.1% SDS) containing phosphorylase and protease inhibitors, 1 mM PMSF. After ultrasonic treatment, the cell precipitation was removed by centrifugation at 12,000 rpm/min. The target protein was enriched with anti-Flag M2 beads (Sigma) or SRSF10 antibody (Santa cruz, sc-101132), and incubated at 4 °C overnight. The immunoprecipitated complexes were washed, and then proteinA/G beads were added on the second day. The immunoprecipitated complexes were subjected to immunoblot analyses with corresponding antibodies. Other antibodies include anti-CK1ε (Abcam, ab207977), anti-GAPDH (Proteintech, 60004-1-Ig), anti-GFP (Proteintech, 50430-2-AP), anti-Flag (Proteintech, 66008-4-Ig), anti-V5 (CST, 13202S), anti-Phospho-(Ser/Thr) Phe (CST, 9631S). The membrane was incubated with ECL Plus Western Blotting Substrate and detected using either X-ray film or Chemiluminescent Imaging System (Tanon 5200).

### Co-IP/MS method

This Co-IP/MS method involves immunoprecipitating the proteins SRSF10 and CK1ε from cell lysates, followed by SDS-PAGE separation, in-gel tryptic digestion, and peptide extraction. The extracted peptides were analyzed *via* nano LC-MS/MS (Ultra High Performance Liquid Chromatography coupled to an Orbitrap Fusion Lumos) using a C18 column and a 5 to 35% acetonitrile gradient. MS data are acquired in data-dependent acquisition mode (top 30 precursors, higher-energy collisional dissociation fragmentation), and phosphorylation sites were validated using phosphoRS.

### Luciferase reporter gene assay

HEK293T cells were transiently transfected with SuperTOPFlash reporter plasmid (0.25 μg) along with control plasmid pCMXβgal (0.05 μg) and the indicated amounts of expression plasmids in six replicates. The transfection was performed using Lipofectamine 2000 (Invitrogen; Thermo Fisher Scientific, Inc.) in appliance with the manufacturer's protocols. Forty-eight hours after transfection, cell lysates were used for luciferase activity assessment with Luciferase assay kit (Promega), and luciferase values were normalized toβ-galactosidase (β-gal) activity.

### Protein purification and peptide synthesis

The expression plasmids for glutathione-*S*-transferase (GST)-tagged CK1ε (GST-CK1ε) were constructed and expressed in *Escherichia coli* (*E. coli*) BL21. The expression of fusion proteins was induced with IPTG (Sigma-Aldrich) at 16 or 28 °C for 8 to 12 h. GST and GST fusion proteins were purified by affinity chromatography with glutathione-sepharose 4B beads (GE Healthcare). The factor Xa protease was used to remove GST tag from fusion proteins. The 266-amino acid polypeptides of SRSF10 WT (1–266 aa), SRSF10 single mutants (S23A, S133A), and double mutant (S23/S133A) were chemically synthesized by QYAO Biotech.

### ADP-Glo kinase assay

White low-volume 384-well polystyrene plates were used for the ADP-Glo kinase assay (Promega, V6930). The 10 μl kinase reaction was carried out in 1x reaction buffer A supplemented with 2 mM MnCl_2_, 2 mM DTT, and 100 μM sodium vanadate. Purified CK1ε kinase was incubated with the following peptide substrates: WT SRSF10, SRSF10 single mutants (S23A, S133A), and SRSF10 double mutant (S23/S133A). Reactions were initiated by adding 10 μM ATP and proceeded at room temperature for 30 min. ADP was detected using 10 μl of ADP-Glo Reagent and 20 μl of Kinase Detection Reagent. Curve fitting was performed using GraphPad Prism software (https://www.graphpad.com/). An ATP-to-ADP conversion curve in the 1 μM series was performed at the same time to correlate the % ADP produced to each relative light unit value.

### Gene expression analysis

Clinical notes from previous studies were mined using R (version 3.2–10) to explore the correlation between SRSF10 and CK1ε expression and patient survival (49). The Survminer R package (version 0.4.9) was used to visualize the survival differences into a Kaplan–Meier plot. For all survival analyses, the median was set as the expressed truncation value, and the HR and *p* value were calculated. A number of functional enrichment analyses were performed using the clusterProfiler R package (version 3.14.3) and org.Hs.eg.db R package (version 3.10.0), including the gene ontology terms [biological processes, cellular components and molecular functions] and the Kyoto Encyclopedia of Genes and Genomes pathway. It is then visualized with the ggplot2 R package (version 3.3.3). Gene set enrichment analysis was conducted based on Hallmarks gene sets (h.all. version. symbols. gmt) extracted from MSigDB database (http://software.broadinstitute.org/gsea/msigdb/index.jsp) to explore the signaling pathways associated with SRSF10’s differentially expressed gene (50).

### Immunofluorescence staining

The tumor tissue was fixed with 4% paraformaldehyde paraffin-embedded and sectioned. Following blocking with 10% goat serum, the samples were incubated with anti-Bcl-xL antibody (CST, 2764S) in blocking buffer (10% goat serum in PBS) overnight at 4 °C and then rinsed and incubated with secondary Alexa Fluor 594–conjugated anti-rabbit antibody (Life Technologies) for 1 h at room temperature. The tissue was then rinsed with PBS, stained with 4,6-diamidino-2-phenylindole, and mounted. The slides were observed with a fluorescence microscope (LSM880, ZEISS).

### Xenograft mouse model

All animal experimental protocols were approved by the Animal Ethics Committee of Shenzhen University (approval no. AEWC-2021006). Male Balb/c nude mice (6 weeks old) were purchased from Guangdong Medical Laboratory Animal Center. The animals were acclimatized to the laboratory for at least 1 week prior to the start of the experiments. Balb/c nude mice were randomly divided into two groups. SRSF10-knockdown A549 cells and the parental counterpart were subcutaneously injected into the right back of the mice (5 × 10^6^ cells per mouse, five mice per group), the growth of tumor was observed and the size of tumor was measured every 2 days. When the tumor volume reached 50 mm^3^, each group of mice was randomly divided into two groups and injected intraperitoneally with 10 mg/kg BW SR3029 or vehicle (10% dimethyl sulfoxide/40% PEG-300/5% Tween-20/45% saline) every 2 days, respectively. Tumor volume was calculated by the formula: 0.528 × length × width^2^. After 7 days of drug treatment, mice were sacrificed, tumors were collected, weighed, and photographed. For protein analyses, tumor tissues were homogenized, and protein was extracted. Western blot assays were performed according to the aforementioned methods. For histological analysis, tumors were fixed in formalin at room temperature for 24 h, embedded in paraffin, and sectioned to 5 μm slices. H&E staining were performed.

### Statistical analysis

The data were statistically analyzed using GraphPad prism 9.0 software. The normal probability plot and variance homogeneity test were used to examine normality of data distributions and homogeneity of variance. Student's *t* test was used to compare the differences between two groups, and the single factor ANOVA was used to evaluate the difference between the means of more than two groups. All results were expressed as mean ± SD. The *p* value < 0.05 was considered statistically significant.

## Data availability

The data that support the findings of this study are available from the corresponding author upon reasonable request.

## Supporting information

This article contains supporting information.

## Conflict of interests

The authors declare that they have no conflicts of interest with the contents of this article.
