# Supplementary material for: CK1ε/SRSF10 axis regulates the alternative splicing of Bcl-x in lung cancer cells
Source: J Biol Chem. 2025 Jul 21;301(9):110508. doi: 10.1016/j.jbc.2025.110508 (PMC12391806; doi:10.1016/j.jbc.2025.110508)
Supplement: Supplementary Figure [file mmc1.docx]

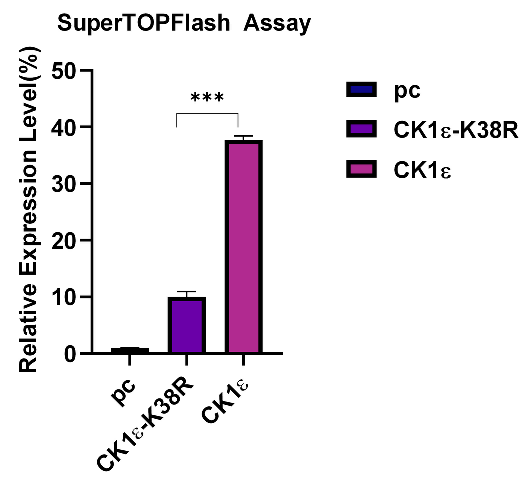


**Supplementary Figure 1. The kinase-dead mutation (K38R) in CK1ε abolishes its kinase activity.** HEK293T cells were co-transfected with SuperTOPFlash reporter and expression plasmids for CK1ε, CK1ε-K38R for 48 h. The luciferase activities were normalized to β-gal signal.


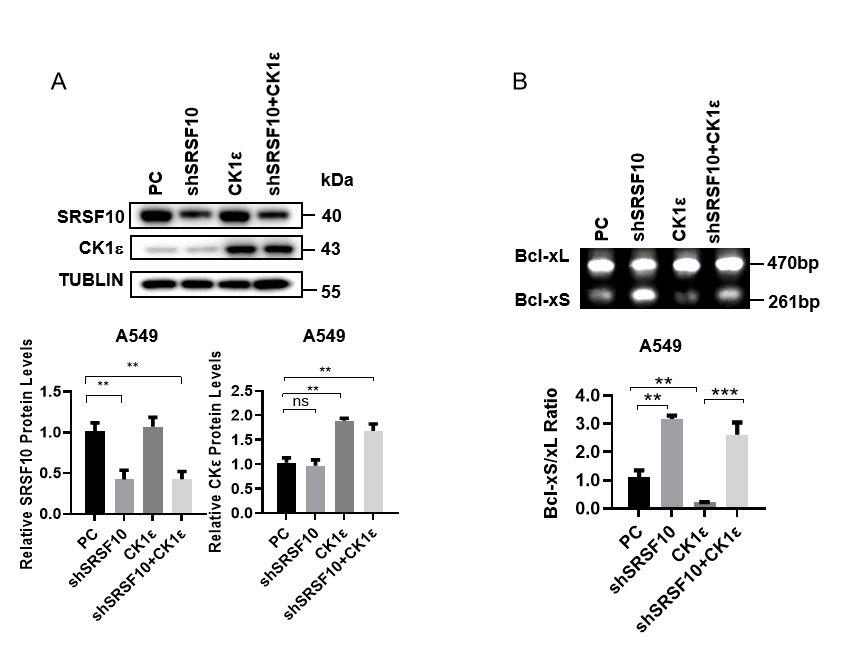


**Supplementary Figure 2.** CK1ε/SRSF10 axis regulates the alternative splicing of Bcl-x in lung cancer cells. (A) The protein expression of CK1ε and SRSF10 in A549 cells with knockdown or overexpression of CK1ε and SRSF10 was detected by Western blot. The protein quantitative results of the grayscale were shown below. (B) CK1ε reversed the effect of SRSF10 knockdown on alternative splicing of Bcl-x in A549 cells. The alternative splicing of Bcl-x was detected by RT-PCR. The quantitative results of the grayscale ratio of Bcl-xS to Bcl-xL are shown below.


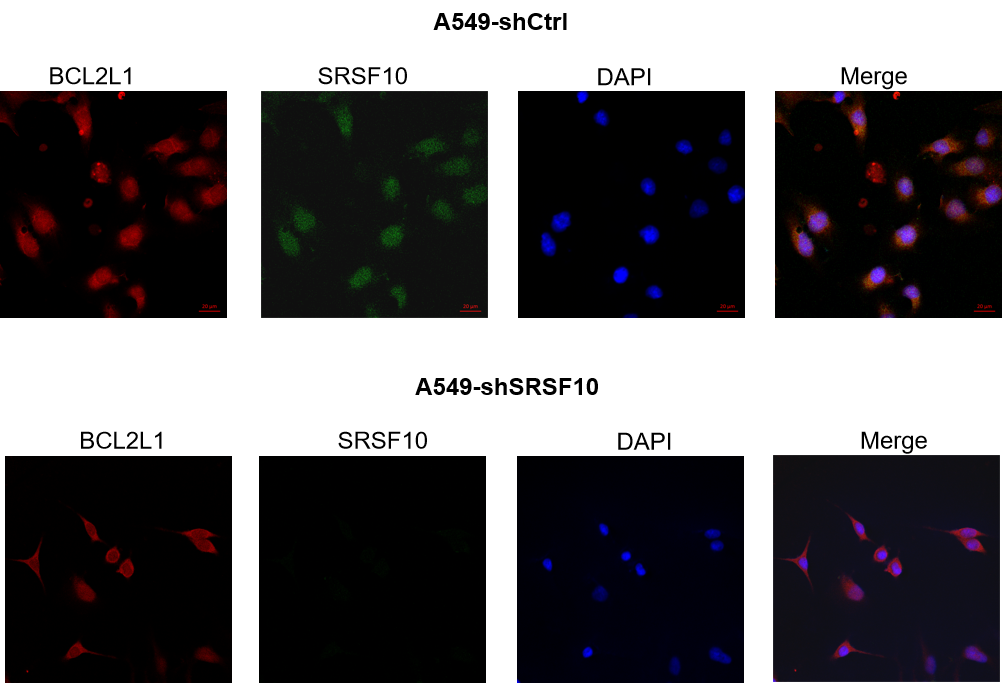


**Supplementary Figure 3. The co-localization of SRSF10 protein and BCL2L1 mRNA in A549 cells with SRSF10 knockdown.** The combination of FISH and IFS methods showed the co-localization of the SRSF10 protein and BCL2L1 mRNA in shCtrl and shSRSF10 A549 cells. Scale bar, 20 μm.


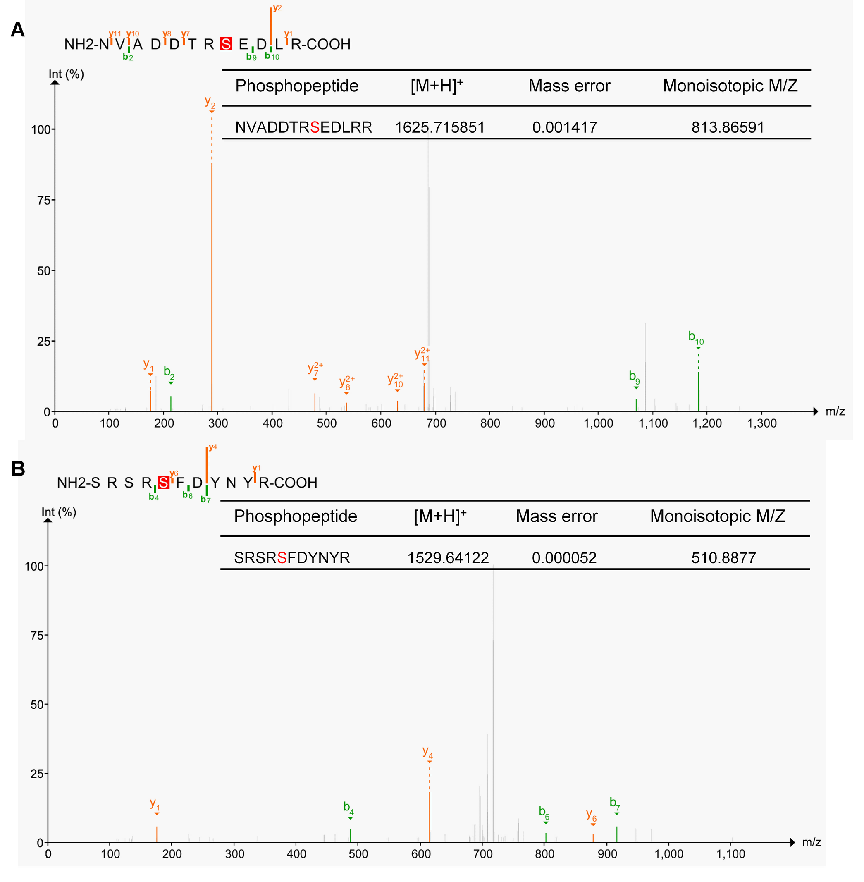


**Supplementary Figure 4. S23 and S133 of SRSF10 are identified as CK1ε-mediated phosphorylation sites.** CK1ε-V5 expression plasmid was co-transfected with expression plasmid for SRSF10-Flag into HEK293T cells. The CK1ε was enriched with anti-Flag M2 beads. The immunoprecipitated complex was subjected to SDS-PAGE separation and visualized by Coomassie staining. The band corresponding to the complex was excised, trypsin-digested, and analyzed by MS.


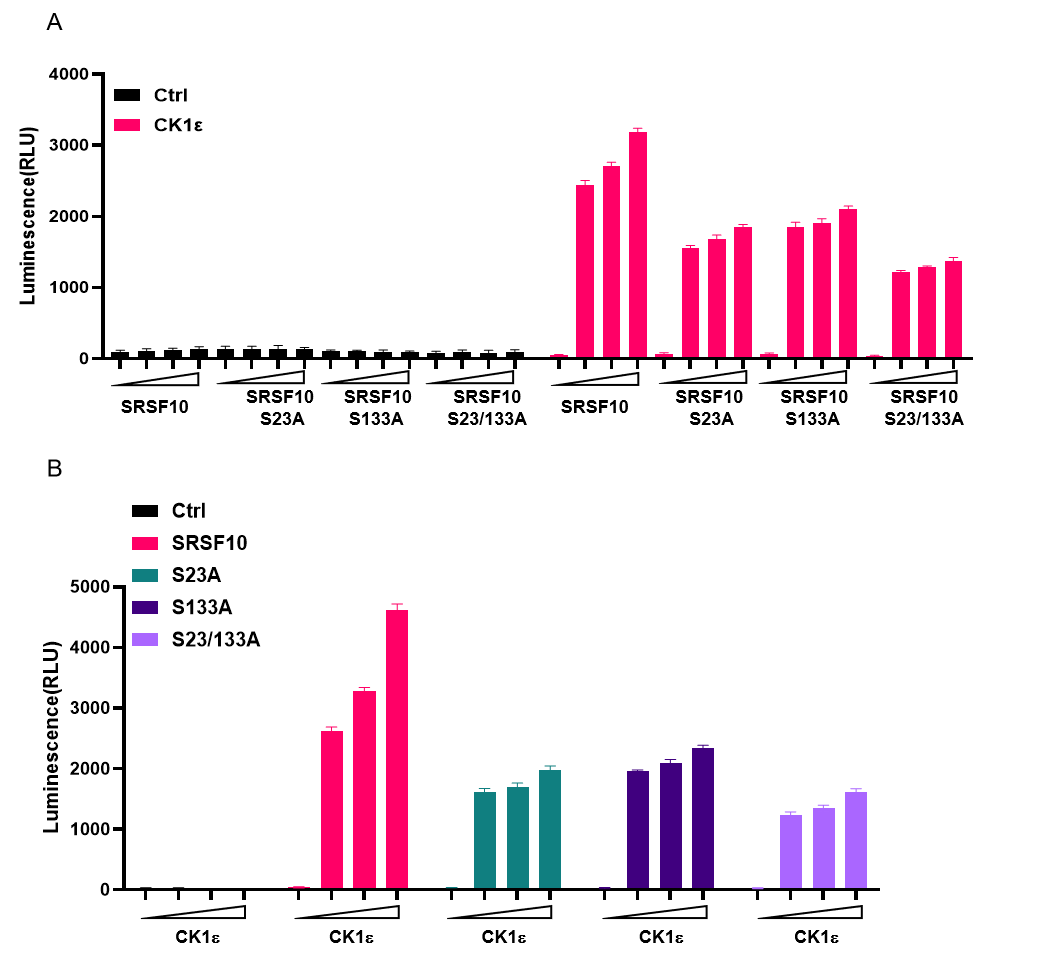


**Supplementary Figure 5. CK1ε-mediated phosphorylation of SRSF10 peptides is significantly reduced in S23/S133 mutant compared to wild-type, as measured by ADP-Glo™ assay.** (A) The synthetic peptide of SRSF10 (1-266 aa), and its mutated peptides (S23A, S133A and S23/133A) at the concentration from 0-500 μM were incubated with the purified CK1ε enzyme 50 μg/μL in a reaction mixture containing 100 μM ATP. (B) The synthetic peptide of SRSF10 (1-266 aa), and its mutated peptides (S23A, S133A and S23/133A) 250 μM were incubated with the purified CK1ε enzyme at the concentration from 0-200 μg/mL in a reaction mixture containing 100 μM ATP. The ATP was measured by detecting the luminescence signal using a microplate reader. The magnitude of the luminescence signal was directly proportional to the CK1ε-mediated phosphorylation of SRSF10 peptides.


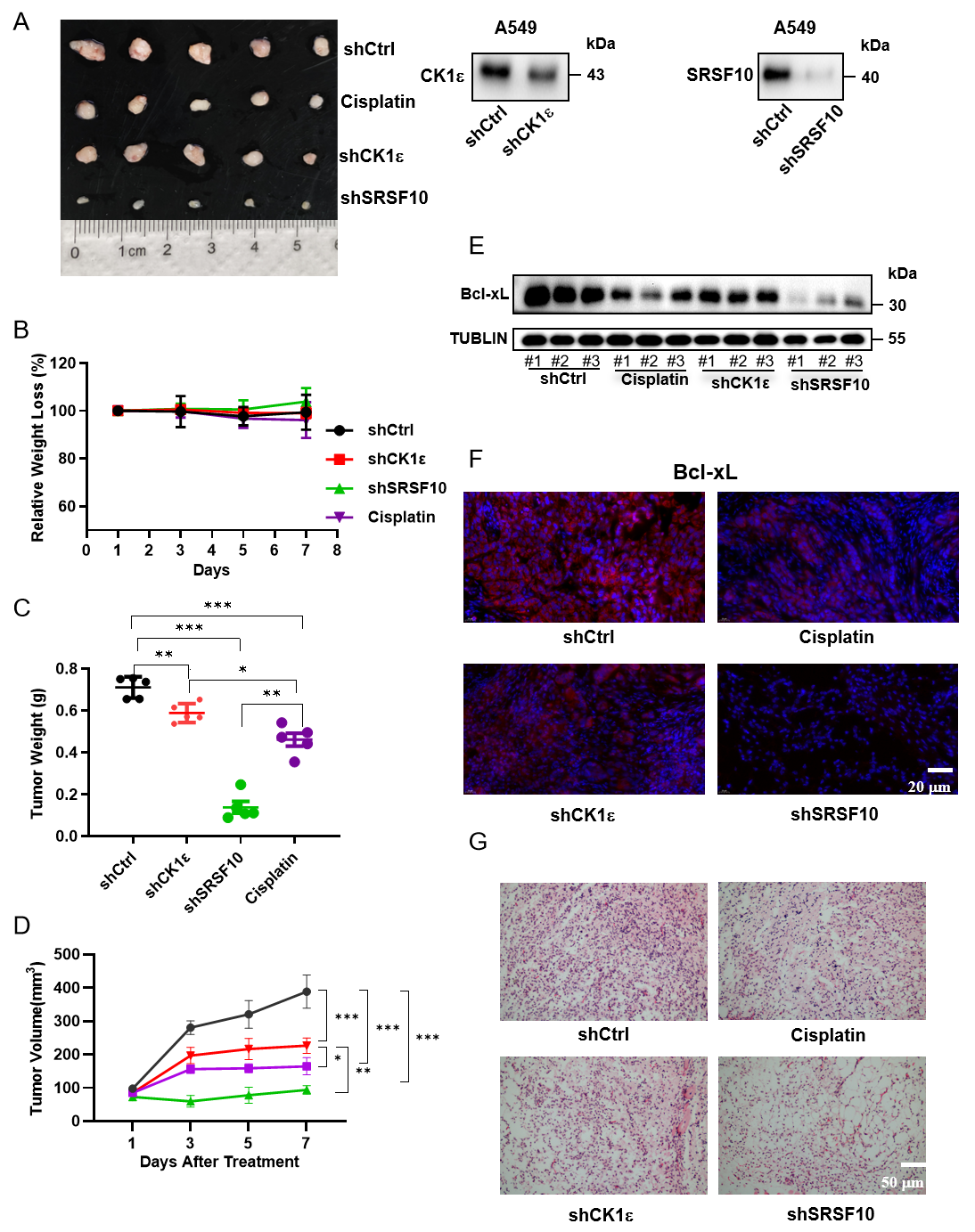


**Supplementary Figure 6. Silencing SRSF10 or CK1ε shows a strong inhibitory effect on lung cancer growth *in vivo*.** (A) The SRSF10-knockdown or CK1ε-knockdown A549 cells and their parental cells A549 shCtrl were subcutaneously (s.c.) implanted into the right back of 7-week-old male Balb/c nude mice to generate a cell line-derived xenograft (CDX) tumor model. The effects of SRSF10 or CK1ε-knockdown in A549 cells were verified by Western blot. When the tumors reached about 50 mm^3^, mice xenografted with A549 shCtrl cells were randomly divided into two groups and intraperitoneally (i.p.) injected with the vehicle or cisplatin (2 mg/kg BW) every 2 days. Tumor sizes were measured with a caliper and tumor volumes were calculated using the formula: 0.528 x length x width^2^. At 7 days after cisplatin treatment, mice were sacrificed, and tumors were collected and photographed. (B) The body weight loss in each experimental group. (C) Mean tumor weight in each experimental group. (D) Mean tumor volume in each experimental group. (F) Immunoblot analysis of Bcl-xL in each experimental group. (F) Immunofluorescence staining of Bcl-xL in each experimental group. Nuclei were counterstained with DAPI. (G) Representative images of H&E staining in each experimental group, scale bars, 50 μm.
